# WHAT IS THE EVIDENCE BASE FOR NONPHARMACEUTICAL INTERVENTIONS TO DECREASE USE OF PSYCHOTROPIC MEDICATIONS?

**DOI:** 10.1093/geroni/igz038.2609

**Published:** 2019-11-08

**Authors:** German Alarcon Garavito, Kathleen Abrahamson

**Affiliations:** 1 Universidad Nacional de Columbia, Bogata, Colombia; 2 Purdue University, West Lafayette, Indiana, United States

## Abstract

Psychotropic medications (anti-depressants, anti-anxiolytics, anti-psychotics, others), have been targeted as a class of medications that can be reduced among residential long-term care (LTC) residents with proper environmental intervention. Reduction of psychotropic medications has the potential to increase resident health/well-being and decrease costs. However, residential LTC facilities face numerous challenges in reducing psychotropic medications, including but not limited to behavior and communication difficulties in advanced dementia, lack of staff training/awareness of needed environmental modifications, and use of medications to manage the cascade of side-effects that occur with polypharmacy and multi-morbidity in the LTC population. A developed literature exists surrounding non-pharmaceutical interventions to reduce psychotropic use in residential LTC. The goal of this current study was to synthesize that body of literature through a systematic review, and categorize findings in a way that is meaningful to direct-care nursing staff. The search of 13 databases identified 14 studies published since 2007 that focused specifically upon residential care and nurse-led non-pharmaceutical interventions. Identified study designs were experimental (5), observational (3), and systematic reviews/meta-analyses from within 8 countries. Findings were categorized based upon type of intervention (educational, environmental, sensorial, care-approach, physiological, and social), as well the target population. Successful interventions were most commonly targeted toward staff and residents, less commonly toward family. The most common approach was education and change in care-approach, with physiological interventions being the least common. We will discuss the implications for nursing staff surrounding the available evidence, and pro/cons of implementing interventions within different organizational settings based upon the study findings.

